# Using a mechanistic framework to model the density of an aquatic parasite *Ceratonova shasta*

**DOI:** 10.7717/peerj.13183

**Published:** 2022-04-14

**Authors:** H. Eve Robinson, Julie D. Alexander, Jerri L. Bartholomew, Sascha L. Hallett, Nicholas J. Hetrick, Russell W. Perry, Nicholas A. Som

**Affiliations:** 1 Arcata Fish and Wildlife Office, U.S. Fish and Wildlife Service, Arcata, CA, United States of America; 2 California State Polytechnic University, Humboldt, Arcata, CA, United States of America; 3 Department of Microbiology, Oregon State University, Corvallis, OR, United States of America; 4 U.S. Geological Survey, Western Fisheries Research Center, Cook, WA, United States of America

**Keywords:** Myxozoan, Actinospore, Klamath River, Salmonids, Disease, Predictive model, Management

## Abstract

*Ceratonova shasta* is a myxozoan parasite endemic to the Pacific Northwest of North America that is linked to low survival rates of juvenile salmonids in some watersheds such as the Klamath River basin. The density of *C. shasta* actinospores in the water column is typically highest in the spring (March–June), and directly influences infection rates for outmigrating juvenile salmonids. Current management approaches require quantities of *C. shasta* density to assess disease risk and estimate survival of juvenile salmonids. Therefore, we developed a model to simulate the density of waterborne *C. shasta* actinospores using a mechanistic framework based on abiotic drivers and informed by empirical data. The model quantified factors that describe the key features of parasite abundance during the period of juvenile salmon outmigration, including the week of initial detection (onset), seasonal pattern of spore density, and peak density of *C. shasta*. Spore onset was simulated by a bio-physical degree-day model using the timing of adult salmon spawning and accumulation of thermal units for parasite development. Normalized spore density was simulated by a quadratic regression model based on a parabolic thermal response with river water temperature. Peak spore density was simulated based on retained explanatory variables in a generalized linear model that included the prevalence of infection in hatchery-origin Chinook juveniles the previous year and the occurrence of flushing flows (≥171 m^3^/s). The final model performed well, closely matched the initial detections (onset) of spores, and explained inter-annual variations for most water years. Our *C. shasta* model has direct applications as a management tool to assess the impact of proposed flow regimes on the parasite, and it can be used for projecting the effects of alternative water management scenarios on disease-induced mortality of juvenile salmonids such as with an altered water temperature regime or with dam removal.

## Introduction

**Figure 1:**
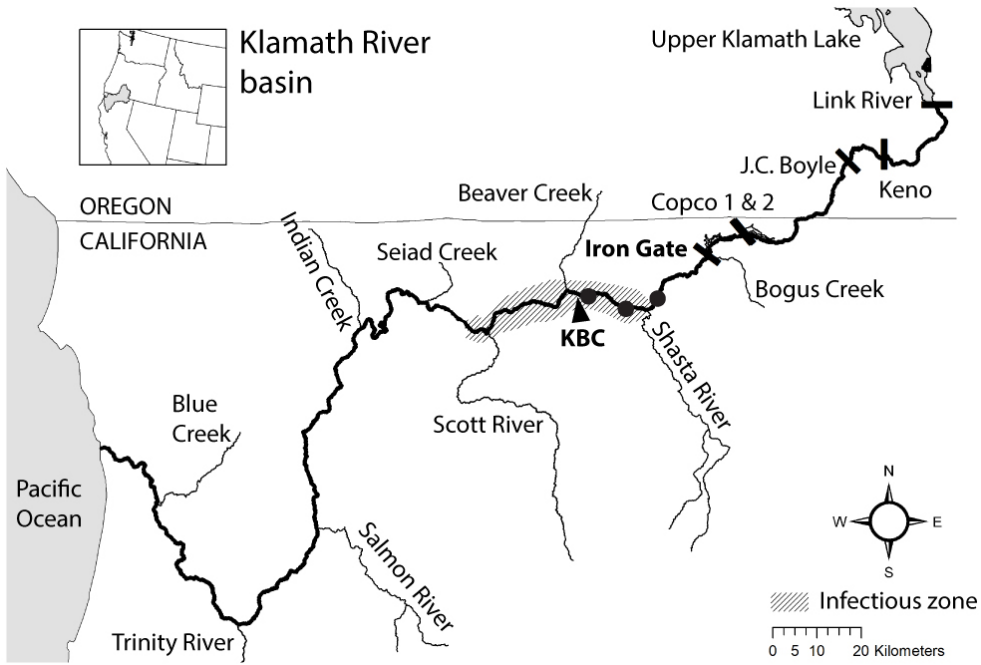
Map of the Klamath River basin in the United States of America. The Klamath River (bold line) runs westward from Upper Klamath Lake to the Pacific Ocean. The map indicates the location of six hydroelectric dams (bars), the infectious zone (shading), the Beaver Creek monitoring site (KBC), and three annelid monitoring sites (circle markers).

Understanding disease dynamics that contribute to population decline is important in the management and recovery of salmonids (*e.g.*, Lehman et al., 2020). *Ceratonova shasta* is a myxozoan parasite that has been the focus of research and management in the Klamath River (USA) because of its effects on the survival rates of juvenile Chinook and Coho salmon (*Oncorhynchus tshawytscha* and *O. kisutch*, respectively; Fujiwara et al., 2011; Hallett et al., 2012). The impact of the parasite on salmonid populations is primarily by infection of juvenile stages, through which disease-caused mortality can reduce year classes of fish. Predicted mortality of juvenile Chinook and Coho salmon can exceed 60% in years when the density of *C. shasta* in the water column is high (*i.e.*, number of parasite spores per liter of river water; Plumb et al., 2019; Som et al., 2019). The Klamath River provides a useful example to investigate the impact of dams on parasite dynamics and how disease monitoring can inform management actions. In the lower Klamath River, outmigrating juvenile Chinook and Coho salmon pass through an “infectious zone”, an area of elevated *C. shasta* densities, located on the mainstem between the confluences of the Shasta and Scott Rivers, downriver from Iron Gate Dam (Hallett & Bartholomew, 2006; Fig. 1). The dam plays an important role in the location of this infectious zone because it limits upstream passage of anadromous fishes and effectively concentrates spawning that facilitates transmission between parasite hosts.

### The lifecycle of *C. shasta*

**Figure 2:**
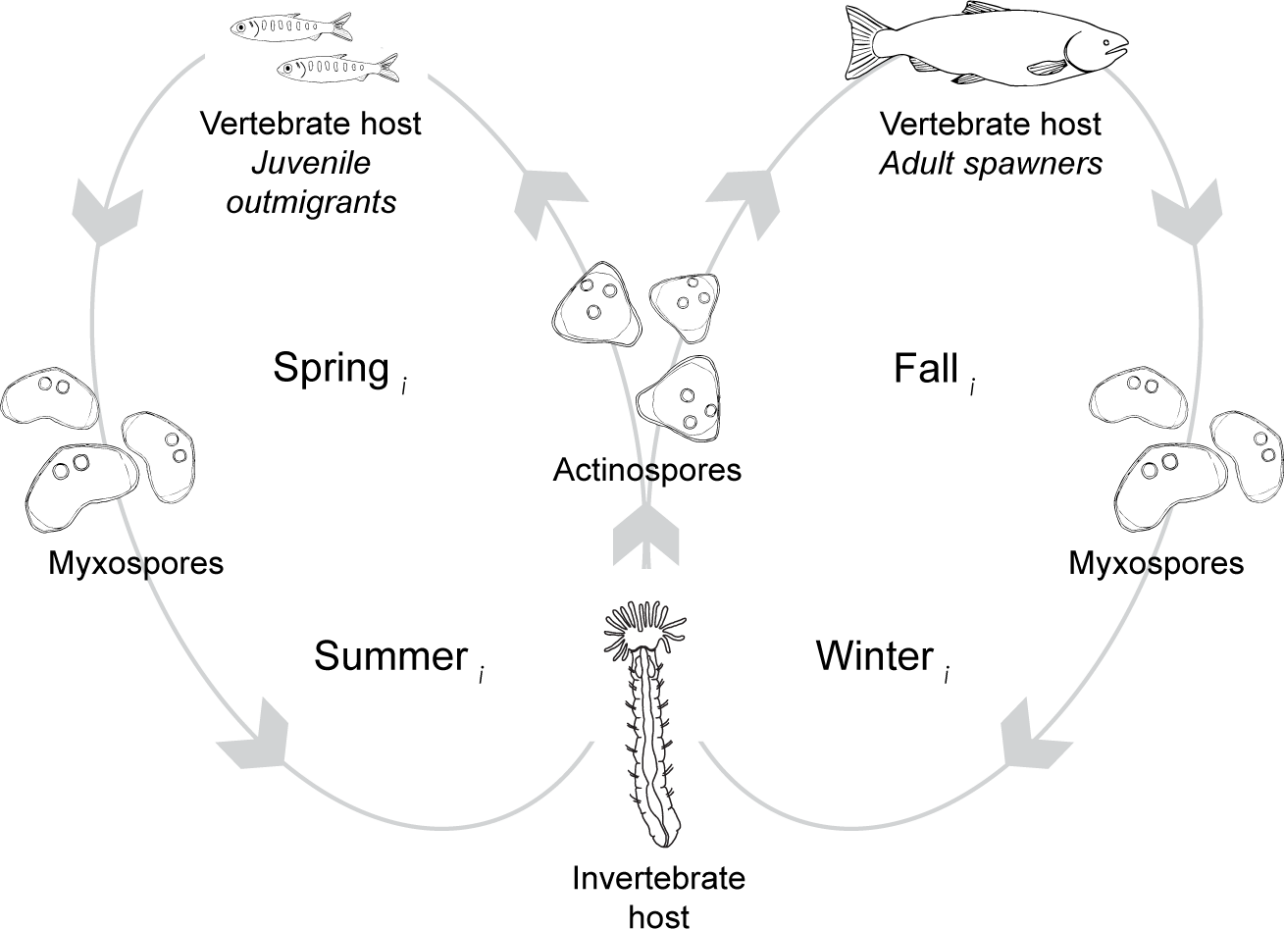
The lifecycle of *Ceratonova shasta* alternates between two spore stages and two hosts, cycling through each phase during different seasons of the year. Actinospore stages infect vertebrate, salmonid hosts (*Oncorhynchus* spp.) and myxospore stages infect invertebrate, fabricid annelid hosts (*Manayunkia occidentalis*). Directional orbits of the lifecycle are represented by gray lines and arrows. In the spring (year = *i*), mature actinospores are released into the water column and encounter outmigrating juvenile salmonid hosts (left loop). Infected juveniles release myxospores that are consumed by annelid hosts. Infected annelids release actinospores in the fall that encounter adult salmonids returning to the river to spawn (right loop). After swimming upriver to spawn, the fish die and release myxospores that can infect annelids. Actinospore development in annelids takes more time in the colder water temperatures of the winter than during the summer. Infected annelids release mature actinospores in spring of the next year (year = *i+1*), and the cycle continues.

*C. shasta* is naturally-occurring in river systems across the Pacific Northwest of North America. The parasite alternates between two waterborne spore stages, actinospores and myxospores, that infect salmonid (actinospores) and annelid (myxospores) hosts (Fig. 2; Bartholomew et al., 1997). Actinospores are released into the water from benthic annelids, *Manayunkia occidentalis* (previously *M. speciosa*; Atkinson, Bartholomew & Rouse, 2020), encounter the fish host, invade through the gills, move into the circulatory system, and proliferate in intestinal tissue causing enteronecrosis, hemorrhaging, and lesions (Bartholomew, 1998; Bjork & Bartholomew, 2010). Infected fish release myxospores that in turn are consumed by annelids. Actinospores develop within infected annelids and are expelled into the water where they can encounter the next host and complete the lifecycle (Bartholomew et al., 1997). In the Klamath River, actinospores released in the spring can encounter and infect outmigrating juvenile salmon; those released in the fall can encounter and infect adult salmon returning to spawn (Fig. 2). Developmental strategies of *C. shasta* differ between infections in adult and juvenile salmonid hosts; in adults, release of myxospores occurs after death (*i.e.*, post-spawn), while in smolts and parr the parasite is released shortly after infection (Kent et al., 2014). The parasite released from infected adult carcasses is dispersed downriver where deposition overlaps with habitat for the invertebrate host.

**Figure 3:**
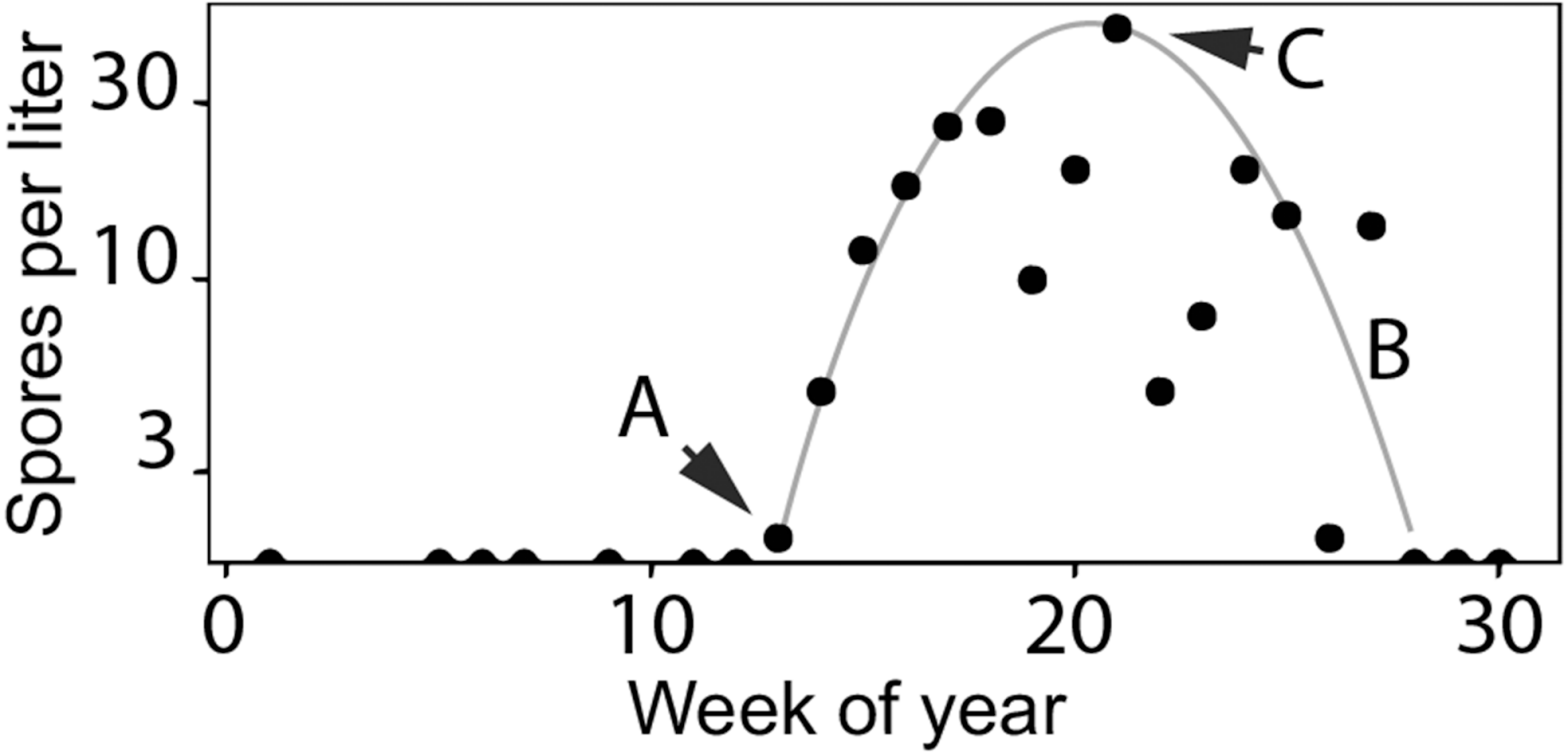
Characteristics of *Ceratonova shasta* densities observed in spring. Spore densities (spores/L) in the spring include (A) the onset of spore release from annelid hosts, (B) the general seasonal pattern of spore density (gray line), and (C) the peak magnitude of spore density. To highlight these features, the black markers shown here are observed values of spore density measured at the Beaver Creek monitoring site in 2014.

### Impacts of *C. shasta* on salmonids

Fish exposed to high doses of *C. shasta* are more susceptible to developing clinical disease (Hallett & Bartholomew, 2012; Ray & Bartholomew, 2013; Ray, Holt & Bartholomew, 2012). Juvenile salmonids outmigrating during the spring season in the lower Klamath River can be exposed to high parasite densities. Actinospore density fluctuates temporally and spatially based on the lifecycle described above (see Fig. 2). As river temperatures warm in the spring season, spore densities increase from undetectable levels to peak levels (see Fig. 3 using 2014 as an example; see Bartholomew et al., 2020 for spore monitoring since 2006). The timing of this increase in spores (the “onset” of spores) impacts infection and disease risk for juvenile salmonids and varies as a function of their migration timing (Kovach et al., 2013) through the infectious zone relative to seasonal variation in spore densities (Perry et al., 2019). The highest densities in the year typically occur in the spring (the “peak” spore density; Bartholomew et al., 2020). For example, migration through the infectious zone of juvenile Chinook Salmon of natural-origin spans March–June (David, Gough & Pinnix, 2018); in some years >80% of the run has outmigrated before the onset of spring spores, thereby minimizing disease risk (Som et al., 2019). In other years, outmigration timing coincides with increases of *C. shasta* densities above levels known to cause high mortality (Ray et al., 2014) likely resulting in the high loss of an entire year class of wild production (Voss, True & Foott, 2018). In addition, approximately five million Chinook Salmon smolts are released from Iron Gate Hatchery (located below Iron Gate Dam, Fig. 1) each year between mid-May and early-June, a period that frequently coincides with peak *C. shasta* densities (Robinson et al., 2020), exposing hatchery-origin fish to potentially high mortality.

The impacts of *C. shasta* on outmigrating salmon have been the focus of various management actions on the Klamath River. Federal agencies manage species listed under the Endangered Species Act including Southern Oregon/Northern California Coast (SONCC) Coho Salmon and southern resident killer whales (*Orcinus orca*) that rely on Chinook Salmon as a primary food source (NMFS, 2019). Flow prescriptions aim to mitigate disease risk to juvenile salmon by disrupting the *C. shasta* lifecycle (NMFS & USFWS, 2013; NMFS, 2019). Flushing flows (≥171 m^3^/s for at least 72 h) have been released from Iron Gate Dam to mobilize sediments and disturb river bed stability (Shea, Hetrick & Som, 2016), thereby reducing the abundance of the annelid host (Stocking & Bartholomew, 2007; Alexander et al., 2014; Alexander et al., 2016).

### Models developed to simulate components of *C. shasta*

A model to simulate the timing and abundance of *C. shasta* in the spring can serve as a critical decision support tool for evaluating the potential effects of flow management scenarios on disease risk of the rearing and outmigration lifestages of juvenile salmon. This model would also be useful in predicting *C. shasta*-caused mortality in existing management assessments such as the juvenile salmon population dynamics model, the Stream Salmonid Simulator (S3; Perry et al., 2019). Incorporating a simulation model for *C. shasta* density into S3 would improve model outputs that currently rely on historical observations of spore density that are insensitive to environmental inputs. Simulating spore density patterns that relate directly to disease risk for outmigrating juvenile salmonids is necessary to both evaluate management scenarios and predict their effects on disease-caused mortality of juvenile salmon. The model needs to include (1) the timing of the initial release of actinospores (“onset”) from the annelid host into the water column, (2) the overall seasonal pattern of spore densities, and (3) the peak magnitude of densities for each year (Figs. 3A, 3B and 3C, respectively).

Models have been developed to quantify some phases of the *C. shasta* lifecycle, providing the foundation upon which to develop a spore density model. A degree-day model using accumulated thermal units (ATUs) predicted the development time of spore stages in hosts (Chiaramonte, 2013; Ray et al., 2015), but this model was based on laboratory experiments and has not been evaluated for accuracy in the development time of *C. shasta* spores in the Klamath River. The degree-day approach is based upon the temperature-dependency of spore production and release and is commonly used in other myxozoan species to characterize spore development in hosts (*e.g.*, *Myxobolus cerebralis*, Kerans, Stevens & Lemmon, 2005; De Buron et al., 2017; *Tetracapsuloides bryosalmonae*, Sudhagar, Kumar & El-Matbouli, 2020). Further, an epidemiological model describing how *C. shasta* dynamics respond to future climate scenarios found that high winter discharge had the greatest impact on lowering the number of infected juvenile fish (Ray et al., 2015), and a statistical model for annelid habitat suitability related hydraulic and substrate variables to annelid host distribution in the Klamath River (Alexander et al., 2016). Other analyses have found a correlative relationship between the prevalence of infection of hatchery-released smolts and the spring peak of spore densities the following year (Robinson et al., 2020), suggesting seasonal links contributing to spore density. This suite of mechanistic and statistical models provides the groundwork to inform simulations of *C. shasta* during the outmigration period.

Understanding the bio-physical linkages in the *C. shasta* lifecycle is important for simulating spore density. A previous attempt to model the abundance of spores in the infectious zone relied on a static population of annelid hosts in a limited section of the river that released actinospores at a constant rate, and the model predicted the prevalence of infection in adult salmon hosts (Schakau, Hilker & Lewis, 2019). However, studies of annelid host populations have documented their range throughout the Klamath River (Stocking & Bartholomew, 2007; Alexander et al., 2014), and laboratory studies have demonstrated spore release rates as highly variable and dependent on temperature and other factors (Bjork & Bartholomew, 2009). In addition, the prevalence of infection in adult salmon is high and not variable, as returning adult fish are immunosuppressed and vulnerable to infection (Dolan et al., 2016); yet a small percentage of carcasses are the highest contributors of myxospores (Foott et al., 2016). As the Schakau, Hilker & Lewis (2019) model predicts infection in adult salmonids, and there is no detectable relationship that links returning spawners with the abundance of spring actinospores (Robinson et al., 2020), a different model design is required to simulate the density of spores in spring that contributes to disease risk in outmigrating juvenile salmonids.

### Study objectives

Our objective was to develop a model to simulate the density of waterborne *C. shasta* during the spring when salmonid outmigration occurs. We aimed to model three key characteristics of spore density patterns that relate directly to disease risk for outmigrating juvenile salmonids. Those characteristics include (1) the timing of the initial release of actinospores (“onset”) from the annelid host into the water column, (2) the seasonal pattern of spore densities, and (3) the peak magnitude of densities for each year (Figs. 3A, 3B and 3C, respectively). Model development was underpinned by data from over a decade of monitoring studies on the Klamath River to explore which biological and environmental components affected these key spore density characteristics. Using these relationships and integrating established mechanistic and statistical relationships, we constructed a model to simulate the density of *C. shasta* during juvenile salmon outmigration that is responsive to changes in environmental conditions subject to resource management decisions.

## Materials & Methods

The Klamath River flows west from Oregon into California (USA; Fig. 1). There are six dams along the Klamath River; the four downstream-most dams are being considered for removal in 2023 (Klamath Hydroelectric Settlement Agreement; see PacifiCorp, 2018). Our study is based on data collected in the infectious zone (ca. 284–230 river kilometers; rkm) downriver from Iron Gate Dam (312 rkm); this dam blocks the migration of anadromous salmon to the upper Klamath River Basin. The Beaver Creek monitoring site lies within the infectious zone (Hallett & Bartholomew, 2006), located just upstream of the confluence with Beaver Creek on the Klamath River mainstem (258 rkm), and is one of the *C. shasta* spore density monitoring stations with the longest period of record. Details of the multiple agencies coordinating data collection can be found in annual monitoring reports (*e.g.*, Bartholomew et al., 2020).

To develop a model that simulates the density of waterborne *C. shasta* in the infectious zone during the outmigration season of juvenile salmonids, we used monitoring data to inform key characteristics of spore density. In the sections below, first we describe how biological and environmental monitoring data were compiled (2006–2018; encompassing January–July, weeks 1–30 each year; ‘Monitoring data’ section). Next, we detail how submodels were constructed to capture the three key features of spore density patterns, including (1) the onset week when *C. shasta* is first detected above 1 spore/L in water samples, (2) the seasonal pattern of spore density, and (3) the magnitude of peak spore density in the spring. Hereafter, these submodel components will be referred to as modules (‘Modules A, B, C’ sections, respectively). Finally, we describe how these modules were combined into a final spore model to simulate weekly densities of *C. shasta* through the spring (‘Spore density model’ section). For each year, the spore model simulates the week of spore release (module A), normalized spore densities (range 0 to 1; module B), and an annual peak spore density (module C). All analyses were conducted using R Statistical Software (R Core Team, 2018).

### Monitoring data compiled for 2006–2018

Multiple monitoring programs collect biological and environmental data on the mainstem of the Klamath River, each with different sampling frequency and methodology described in subsections below (‘Biological variables, ‘Environmental variables’; Table 1). Variables were carefully selected based on the hypothesized potential to influence the lifecycle of *C. shasta*.

**Table 1:** Biological and environmental variables summarized for each year 2006–2018. The number of adult Chinook Salmon returning to spawn (*i.e.* fish escapement) were averaged by week in mainstem reaches upstream of the Beaver Creek monitoring site (Gough et al., 2020). The density and prevalence of infection (POI) of the annelid host, *Manayunkia occidentalis*, is averaged from monitoring sites upstream of the infectious zone for 2010–2018 (Alexander et al., 2014). Daily river discharge was extracted from a U.S. Geological Survey gage (station 11516530; USGS, 2019) at Iron Gate Dam. Daily river water temperature at the Beaver Creek site was estimated by the RBM10 model (Perry et al., 2011). Seasonal summaries of “spring” included data from February–April. Reservoir storage and precipitation levels in the Klamath River Basin were extracted from May 1 summaries issued by the California Department of Water Resources (CDWR 2006–2018).

		Units	Abbreviation	Data sources
*Biological*				
	fish escapement	#/week	Esc	Gough et al. (2020)
	annelid density^a^	#/m^3^	Mo.dens	Alexander et al. (2014)
	annelid POI^a^	%	Mo.poi	Alexander et al. (2014)
*Environmental*				
	flow spring peak	m^3^/s	Q.sppeak	USGS gage at Iron Gate Dam
	flow spring sum	m^3^/s	Q.spsum	USGS gage at Iron Gate Dam
	flushing flow	≥171 m^3^/s	FF	USGS gage at Iron Gate Dam
	temperature spring peak	°C	T.sppeak	RBM10; (Perry et al., 2011)
	temperature spring sum	°C	T.spsum	RBM10; (Perry et al., 2011)
	reservoir storage level	% 30-yr average	reserv	CDWR 2006–2018
	precipitation level	% 30-yr average	precip	CDWR 2006–2018

**Notes.**

a Monitoring data available for years 2010–2018.

#### Biological variables

Measurements of the density of waterborne *C. shasta* actinospores (‘spores’ hereafter) were included in this study to examine relationships with biological and environmental variables in constructing a model to simulate spore density, and then used to assess the goodness-of-fit. Spore density at the Beaver Creek monitoring site is measured as part of a long-term monitoring program on the mainstem of the Klamath River (Bartholomew et al., 2020). Water samples were collected by manual grabs (2006 and 2007) or by an automatic sampler (Teledyne ISCO 3700; 2008–2018). In weeks when collection from the automatic sampler was not possible, grab samples were collected. Three 1-L water samples were collected per sampling date at a range of frequencies and time periods: once every two weeks in 2006 (January–July) and 2007 (May–September), twice a week in 2008 (March–September) and 2009 (April–December), and weekly throughout the entire year from 2010–2018. The amount of *C. shasta*- deoxyribonucleic acid (DNA) in each sample was measured using established quantitative polymerase chain reaction (qPCR) protocols and converted to spore density using standard curves (Hallett & Bartholomew, 2006; Hallett & Bartholomew, 2012). Samples with high inhibition (*e.g.*, ineffective qPCR) were excluded, so not all sample dates retained three replicate measurements. For each sample date, the average and range of spore density was calculated and the method of collection (automated sampler or grab) was identified. Peak spore densities were calculated as the maximum density of *C. shasta* spores observed annually and in the “spring” (February–June).

Spore density measurements reflect abundance of the waterborne parasite that includes all spore stages (actinospores and myxospores) and genotypes (0, I, and II). Though molecular assays cannot distinguish between actinospores and myxospores, sentinel studies in which fish tested positive for *C. shasta* after exposure to river water demonstrate the spring pulse is primarily actinospores (Stocking et al., 2006; Hallett & Bartholomew, 2012). The proportion of *C. shasta* genotypes can be measured in water samples; the genotypes associated with mortality in Coho Salmon and steelhead (genotypes II and 0, respectively) comprise a small proportion of total spore density (Atkinson & Bartholomew, 2010). The genotype (I) associated with mortality in Chinook Salmon typically comprises the highest proportion (>80%; Atkinson & Bartholomew, 2010; Hallett & Bartholomew, 2012), therefore total spore density used in this study correlates with genotype I. Though observations of the fish host in this study focus on Chinook Salmon, the total spore density encompasses disease risk for all salmonids.

Estimates of adult Chinook Salmon returning to spawn in the fall were extracted from reports that summarize annual escapement in a long-term Klamath River monitoring program (2006–2018; Gough et al., 2020). Weekly estimates were sums of the abundance of spawners in mainstem reaches upstream of the Beaver Creek monitoring site (Beaver Creek to Iron Gate Dam), based on redd counts and mark-recapture of spatially-referenced carcasses. These weekly-stratified estimates were used to calculate run-timing and summed for each year for annual escapement.

Long-term monitoring of the relative abundance of the invertebrate host *M. occidentalis* (individuals/m^3^) was measured at three index sites on the Klamath River mainstem (Fig. 1; circle markers): a site adjacent to the Beaver Creek monitoring site, and upstream sites near the Tree of Heaven campground (281 rkm) and near the intersection with the Interstate-5 freeway (294 rkm). These monitoring sites have persistent annelid populations and provide a reliable index of the host population that releases actinospores entering the infectious zone (Alexander et al., 2014). Three replicate samples were collected at each site according to methods detailed in Alexander et al. (2014) and returned to the laboratory for processing (J.L. Fryer Aquatic Animal Health Laboratory, Oregon State University, Corvallis, Oregon, USA). Densities of annelids (individuals/m^3^) and the prevalence of infection in annelids (%) were calculated for each site and averaged for each sample date. The monitoring data collected early in the year (before spore release) were most relevant to *C. shasta* densities in the spring and were available for 2010–2018.

#### Environmental variables

Daily averages of river discharge were extracted from the historical data of US Geological Survey (USGS) gage 11516530 at Iron Gate Dam USGS, 2019. Daily averages of temperature were estimated near the Beaver Creek site by the River Basin Model-10 water temperature model (RBM10, Perry et al., 2011). Seasonal and annual metrics were also summarized. For each year, daily discharge and water temperature were calculated as a seasonal sum for “spring” (February–April) to indicate warmer or wetter springs. This seasonal index encompassed a narrower range of months than for biological factors to reflect conditions early in the year that affect the parasite. Similarly, seasonal peaks of discharge and temperature were identified as maxima for the spring. For each year 2006–2018, a binary categorical variable was generated to indicate whether a flushing flow event (FF; discharge event reaching at least 171 m^3^/s for 72 h NMFS, 2019) occurred or not during the spring. Additional variables that impact river flow and water temperature (annual reservoir storage and precipitation levels) were extracted from May 1 annual summaries issued by California Department of Water Resources CDWR (2006) that represent the percentage of the 30-year average on May 1. Reservoir storage levels were included to provide a measure of cumulative water availability; low levels indicate previous years of drought, while high levels indicate consecutive years of having a relatively abundant water supply. Levels of precipitation were included as an indicator of whether the given year was relatively wet or dry.

### Module A: simulating spore onset using a bio-physical degree-day model

Simulating the initial release date of actinospores each spring relied on coupling parameterized attributes of the parasite lifecycle with monitoring data (salmonid escapement, water temperature, and the occurrence of high flow events) and results from laboratory studies (spore development within annelids). Laboratory experiments demonstrated that following the requisite ATU for spore development (830 ATU; Meaders & Hendrickson, 2009; Alexander et al., 2015), and despite the detection of mature actinospores in infected annelids, release occurred only after temperatures increased above 10 °C (J. Alexander, 2019, unpublished data). To implement these laboratory findings in the module to simulate spore release, both degree-day accumulations and water temperature were included. Given that Klamath River annelids become infected during the fall or winter with myxospores released by the carcasses of adult salmon, in constructing this module, the degree day accumulations were initiated at the beginning of the week when escapement reached the 25th percentile for each year. The 25th percentile of escapement approximates when spawning is underway, the first spawners have died, and myxospore deposition has begun. In the model we assumed after >830 ATU were accumulated and average weekly water temperature ≥11 °C, spores were potentially detectable in water samples; yet for years in which flushing flows occurred, spore release was delayed for 3 weeks after water flow receded below the threshold for flushing flows (Bureau of Reclamation BOR, 2018; Som et al., 2019). Hence, the simulated week of spore release was determined by the timing of adult escapement and spore development, when water temperatures reached at least 11 °C, and whether a flushing flow occurred for 2007–2018. Spawning occurs in the previous calendar year so model output for 2006 was not available. An assessment of the goodness-of-fit of module A compared the simulated week of spore onset to the observed week of spore release from monitoring data. We used a correlation coefficient to assess the strength and direction of the relationship for this and all subsequent goodness-of-fit assessments. We calculated a Pearson correlation coefficient (*r*) using the *cor.test* function in R Statistical Software (method = “pearson”; R Core Team, 2018). This comparison excluded 2007 and 2009, when monitoring of spore density started after the onset of spores and therefore the observed timing of release was not available.

### Module B: simulating spore density using a temperature-dependent quadratic model

A parabolic thermal response has been described for spore densities based on monitoring studies whereby spore density increases with temperature up to a threshold beyond which spore density declines with further temperature increases (see Perry et al., 2019). The temperature dependency of parasites has been described in other parasite systems with invertebrate hosts by a quadratic relationship between water temperature and abundance (Mangal, Paterson & Fenton, 2008; Paull, LaFonte & Johnson, 2012; Mordecai et al., 2013; Mordecai et al., 2017). Temperature-dependent processes in the lifecycle of *C. shasta* include the development of spores within hosts (see module A) and also the viability of spore stages in the water once released (Bjork & Bartholomew, 2010). To quantify the independent thermal response isolated from confounding factors, we excluded years in which flushing flow events occurred, as removing hosts by scouring annelids disrupts this relationship, and those in which there was no signal of spore density (selected years: 2008, 2010, 2014, and 2015). The occurrence of flushing flows and low spore density are addressed in module C. The isolated thermal response is important to describe the overall pattern of spore density increase and decrease in response to temperature. Spore densities were normalized to a common relative scale (0 to 1) by dividing spore densities in each year by the annual peak density. A quadratic regression model was then fit to the normalized spore densities with corresponding daily temperature and temperature squared as explanatory variables for the selected years. Using normalized spore densities in the model focuses on describing the shape of the thermal response. Graphical diagnostics were used to evaluate the assumptions of the model; parameters were estimated using the *lm* function in R (R Core Team, 2018). A goodness-of-fit assessment for module B compared simulated to observed spore densities for years not included in the quadratic regression model. The estimates of the quadratic model were applied to daily temperatures of each year starting at the onset (module A) to simulate normalized spore density.

### Module C: simulating peak spore density using a generalized linear model

To simulate peak spore density, we used a generalized linear model (GLM) appropriate for the distribution of spore density data and built upon a published relationship that correlated peak spore density with the prevalence of infection of hatchery-origin Chinook Salmon smolts (HPOI, Robinson et al., 2020). The module framework is described below, followed by model selection and fit.

Spore density data were modeled as generalized linear models with a logarithm link function (Faraway, 2006) to account for the observed mean–variance relationship, whereby variability in spore density increases with mean spore density. We used the negative binomial distribution to account for overdispersion that was evident from fitting Poisson GLMs (Zuur et al., 2009). We assume that peak spore density each year (*Y*_*i*_; where *i* represents year) arises from a negative binomial distribution (*NB*) with mean parameter (µ_*i*_) and dispersion parameter (*k*):

\begin{eqnarray*}{Y}_{i}\sim NB \left( {\mu }_{i},k \right) \end{eqnarray*}

The effect of covariates on spore density was modeled though a logarithmic function that links a linear function of parameters and explanatory variables to the mean of the distribution:

\begin{eqnarray*}\log \nolimits \left( {\mu }_{i} \right) =X\beta \end{eqnarray*}

where *X* is a design matrix containing explanatory variable values and *β* is a vector including an intercept and regression parameter values. We used maximum likelihood techniques to fit models and estimate parameter values using the *glm.nb* function from the R Statistical Software *MASS* library (R Core Team, 2018).

Building upon the previously described relationship between peak spore density and the prevalence of infection of hatchery-origin Chinook Salmon smolts (HPOI, Robinson et al., 2020), we evaluated other potential biological and environmental covariates (Table 1) to further explain variation in peak spore density in the spring. The selected variables were limited to those supported by ecological hypotheses. Due to the relatively small number of years available for this analysis, we tested the improvement in model fit provided by adding each selected variable with HPOI against the previous model containing only HPOI. This was done using likelihood ratio tests, which are appropriate for testing among nested models (Lewis, Butler & Gilbert, 2011). The likelihood ratio test is a more conservative choice by providing a formal statistical test of each variable’s improvement in model fit rather than model selection based on the number of units from an Akaike Information Criterion (AIC)-type model selection procedure (see Fig. 1 in Lewis, Butler & Gilbert, 2011). It was decided, *a priori*, that no models including interaction effects were considered to avoid over parameterizing models given the limited data availability. For each year, peak spore density was simulated by the best model fit. Module C was assessed by a goodness-of-fit comparison between the simulated and observed peak spore density from monitoring data for each year.

### Spore density model

The spore density model combines output from modules A, B, and C detailed above. Our goal was to simulate the density of *C. shasta* spores in the infectious zone through the period of outmigration for juvenile salmonids. The spore density model generates daily values that are averaged for each week 1–30 in 2007–2018; the model terminates at week 30 when most juvenile Chinook Salmon outmigrants have passed downstream of the infectious zone (David et al., 2017), and simulation results for 2006 are not available as the model requires inputs from the previous year. For each year the model combines output from modules A, B, and C. First, spore density is set to zero each week at the beginning of the year before the onset of spore release. The week of spore release is simulated by the bio-physical degree-day module (A). After onset, normalized spore densities (range from 0 to 1) are simulated by the quadratic regression module (B). This module provides the overall seasonal spore density pattern, while the magnitude of spore density for years in which flushing flows occur is simulated by the linear module (C). Last, simulated spore densities are calculated by multiplying the simulated normalized spore density by the simulated peak spore density. A goodness-of-fit comparison between simulated and observed values used the simulated densities for days in which spore density was measured. Simulated spore density was averaged by week.

## Results

**Figure 4:**
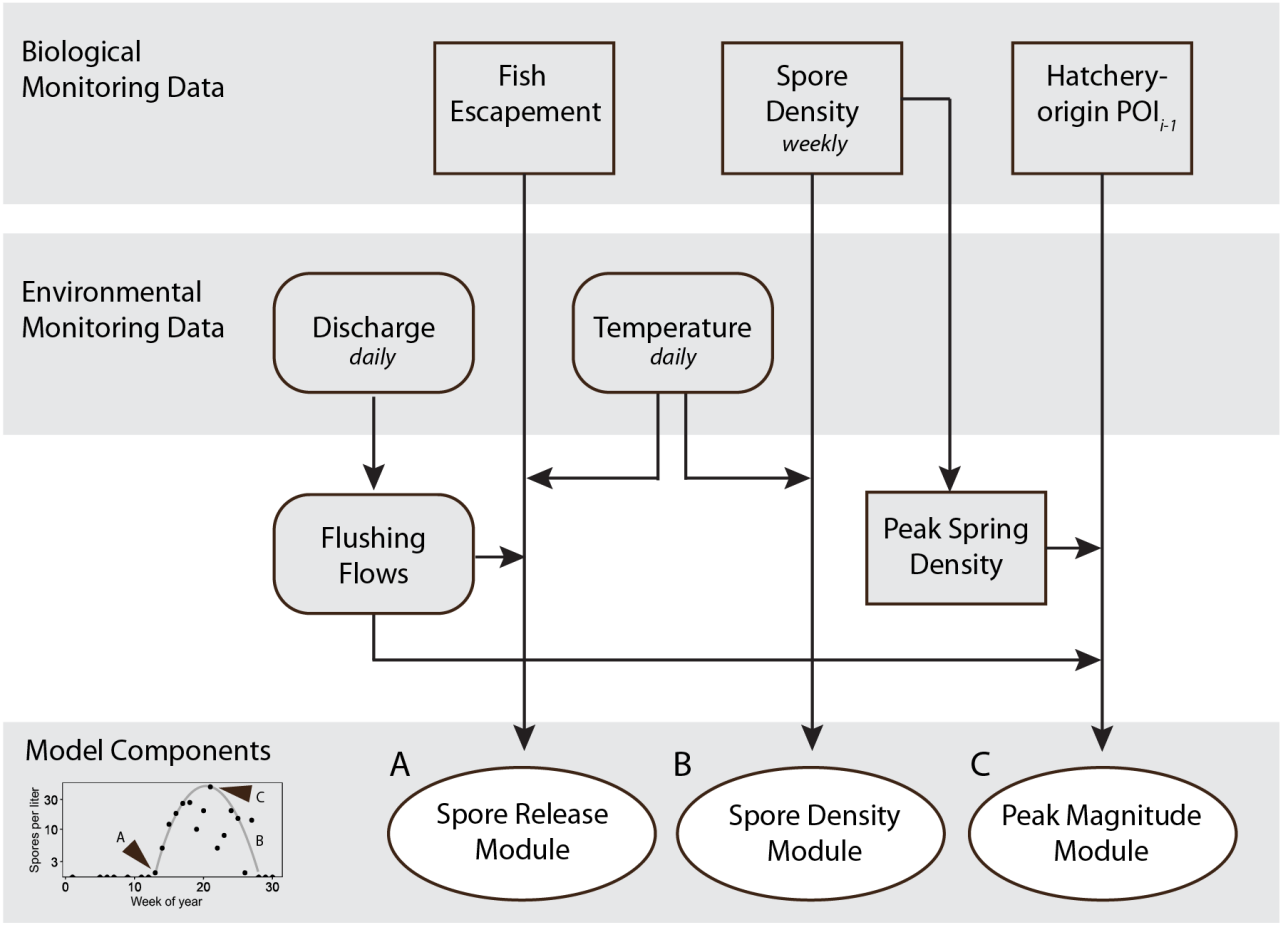
Schematic showing monitoring data included in the final spore model for *Ceratonova shasta* that predicts spore density. Modules predict the onset of spore release from annelid hosts (A), spore density increase with water temperature (B), and peak magnitude of spore density (C). Inputs for the modules are biological monitoring data (adult Chinook Salmon returning to spawn, *i.e.*, escapement, density of spores in water samples, the prevalence of infection of hatchery-origin juvenile Chinook from the previous spring [POI_*i*−1_]) and environmental monitoring data (river discharge and water temperature) data shown in the top gray shaded bars. The bottom-left graph (from Fig. 3) shows an example of the three components of spore density that this model predicts (monitoring data from 2014). Predictions generated by each module are validated against observations of spore densities from monitoring data (Figs. 5–7). Arrow direction indicates the order of calculations and analyses.

The model simulating spore density of *C. shasta* in the spring during the outmigration of juvenile salmonids includes three modules that each describe a key characteristic of spore density patterns (illustrated in Fig. 4). Results of the modules are presented first, then followed by results of the complete spore model.

**Figure 5:**
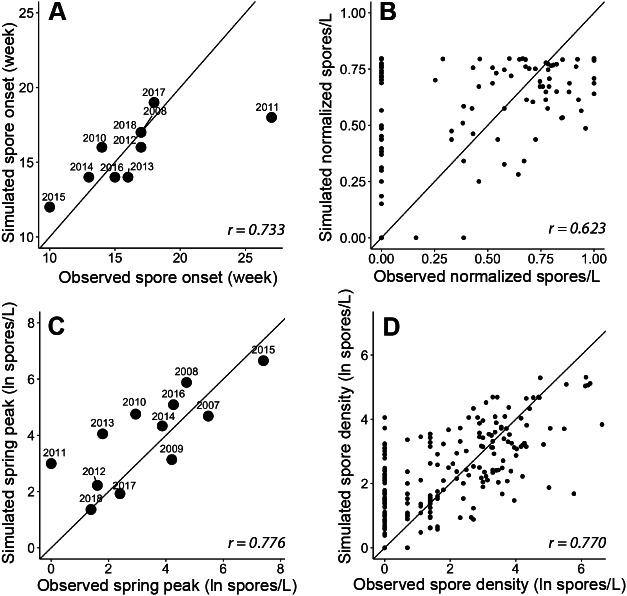
Goodness-of-fit for each module and the final spore model that simulate characteristics of *Ceratonova shasta* spore density. On each panel A–D, the solid line represents a 1-to-1 relationship and the Pearson correlation coefficient (*r*) is reported in the bottom right. (A) The week of spore release simulated for each year by module A compared to observations of initial spore detection (spores/L ≥ 1). The observed week of spore release was not available for 2007 and 2009 as spore monitoring started when spore density was already above 1 spore/L (post-onset). (B) The normalized spore density values simulated by module B compared to observed spore density (normalized by dividing by the maximum annual spore density to range from 0 to 1). Only years excluded from developing the quadratic thermal response were used in this assessment of model fit. (C) The annual spring peak of spore density (natural log of spores/L) simulated by module C compared to observed peak spore density for each year. The selected model simulates the seasonal maximum of spores based on the prevalence of infection of hatchery-origin Chinook juveniles from the previous year (HPOI) and the occurrence of a flushing flow (FF; see Table 3). (D) Spore density (natural log spores/L) simulated by the final spore model compared to observed spore density for 2007–2018.

### Module A: simulating spore onset using a bio-physical degree-day model

The simulated onset of spore release in the spring closely matched observations of initial spore detection from monitoring data (Fig. 5A; *r* = 0.733). For each year, the module started on the average week when escapement reached the 25th percentile, which was week 45 (SD = 0.6). The accumulation of 830 ATU (spore maturity) was reached on average on week 10 of the following year (SD = 2.0). In all cases, this occurred before spores were detected in water samples, which suggests that factor(s) in addition to accumulated temperature influence the timing of spore release. When we included the minimum temperature threshold and delay due to flushing flows, the simulated week of spore release aligned with the observed week of spore release. For 2011, in which observed spore densities remained near zero for the entire spring, the model simulated spore release earlier than the observed week when spores reached 1 spore/L (Fig. 5A), however the model simulated a relatively late week of release. In other years of available data, the week of simulated spore release and observed spore release were similar (mean difference = 0.20 weeks, SD = 1.3).

### Module B: simulating spore density using a temperature-dependent quadratic model

**Figure 6:**
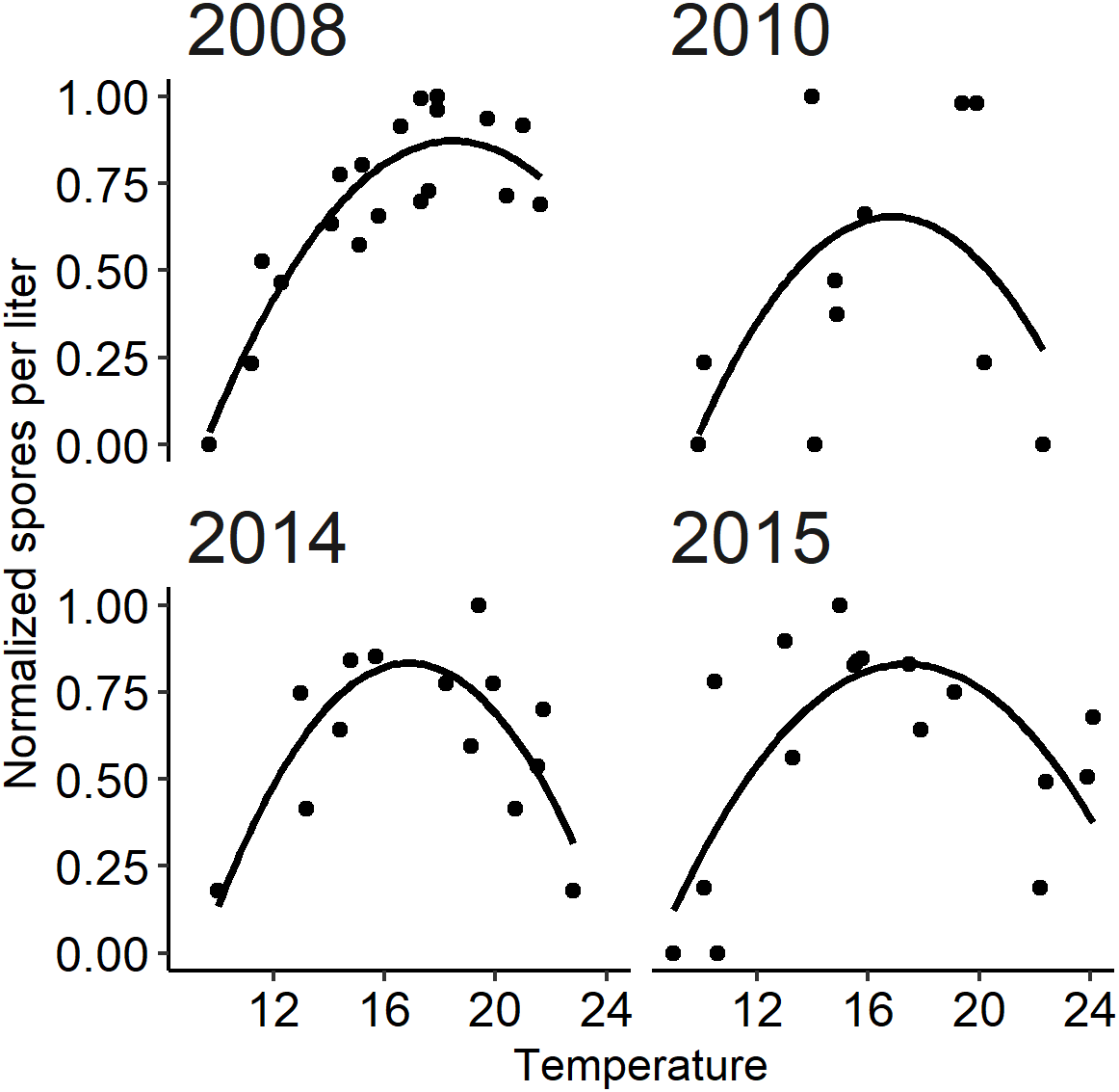
The relationship between observed *Ceratonova shasta* spore density (spores/L) and water temperature (°C) during spring months (February–June) fit with a quadratic curve. Spore density values in each year were normalized by dividing by the maximum annual spore density to range from 0 to 1. The selected years (2008, 2010, 2014, and 2015) met the criteria of having spore densities >1 spore/L, and no flushing flow ≥171 m^3^/s for 72 h.

The quadratic regression model of spore density and water temperature was based on selected years that did not experience a flushing flow event and that detected spore densities (>1 spore/L; Fig. 6). A quadratic curve fit the non-linear relationship between normalized spore density and water temperature, revealing strong evidence (*P* < 0.001) that both temperature variables were associated with seasonal spore density (Table 2). There was no evidence of deviation from the assumptions of the model. Goodness-of-fit model assessment compared simulated and observed spore densities (normalized) for years excluded from the development of the quadratic model (Fig. 5B; *r* = 0.623). Applying the quadratic relationship to all years, daily water temperatures were used to simulate normalized spore density (ranging from 0 to 1) for each day in the spring starting with the week of simulated spore onset (output from module A), and averaged by week.

**Figure 7:**
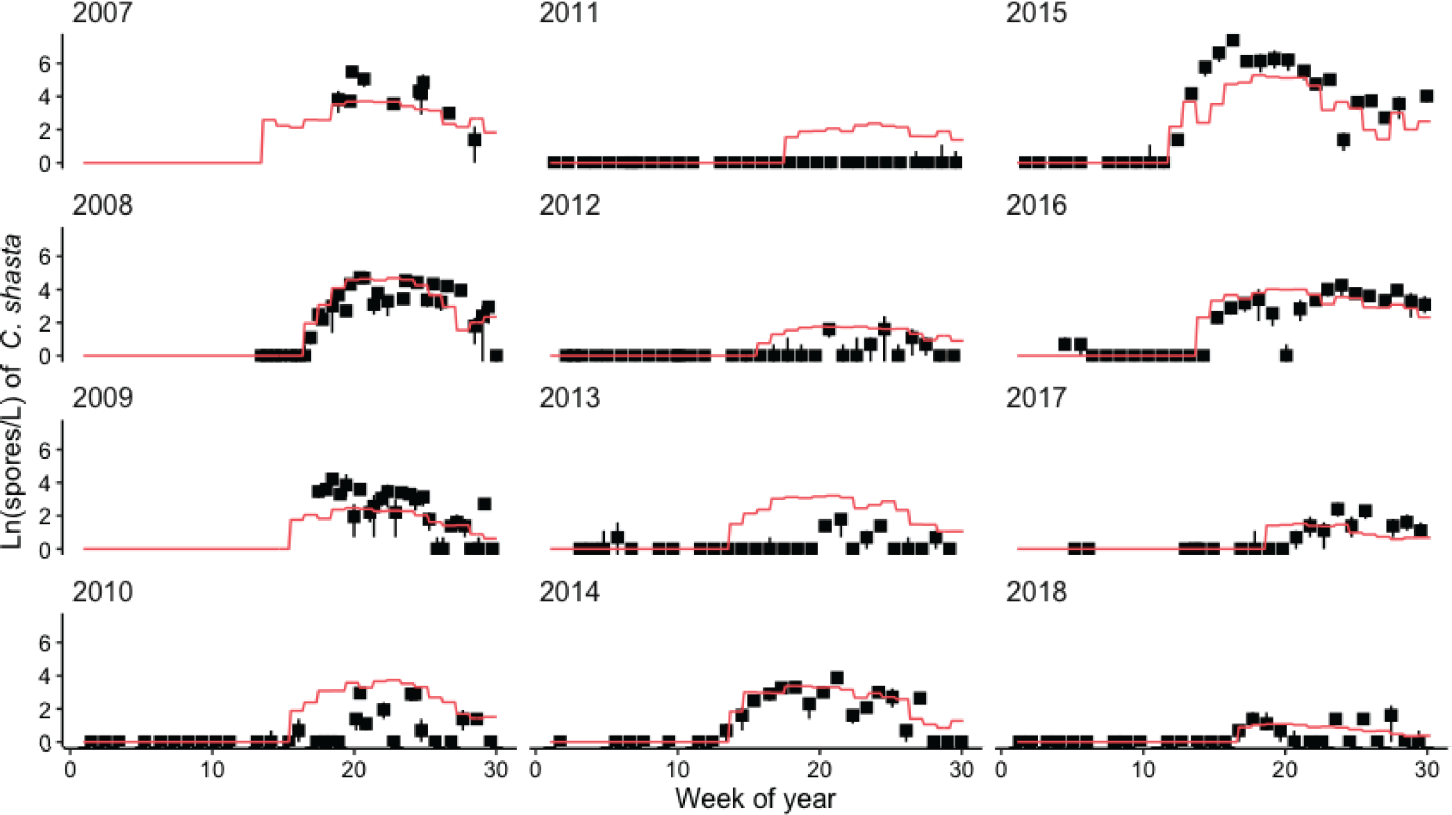
Model simulating spore densities of *Ceratonova shasta* for weeks 1–30 in years 2007–2018. Simulated spore densities (ln spores/L; orange line) were generated by the spore model that combined modules A, B, and C. Observed spore densities are displayed for model assessment. The range of observed spore density values (black lines; min–max) and mean (black squares) are based on replicate water samples measured at the Beaver Creek monitoring site.

### Module C: simulating peak spore density using a generalized linear model

We analyzed improvements in model fit with the addition of biological and environmental variables that potentially influence the lifecycle of *C. shasta* (Table 1). Monitoring data for annelid density and prevalence of infection were available for fewer years than spore densities at the time of this study, which would limit the power of analyses. Therefore, these data were not included in subsequent model fit analyses yet should be considered in future years when more monitoring data are available. Potential variables represent the spring conditions of discharge, water temperature, precipitation, reservoir level, and flushing flows. After implementing likelihood-ratio tests and the resulting *χ*^2^ statistics, a model containing the prevalence of infection of hatchery-origin fish from the previous year (HPOI) with the occurrence of flushing flows (FF) was selected. For most other variables considered, there was no evidence of improved model fit (Table 3). There was weak-to-moderate evidence that annual precipitation improved model fit (*P* = 0.065), but stronger evidence (*P* = 0.020) that the flushing flow events improved model fit (Table 3). Given these results, the model containing HPOI and the occurrence of flushing flows successfully simulated the magnitude of peak density for each spring (Fig. 5C; *r* = 0.776).

**Table 2:** Results of the quadratic model between spore density and water temperature. The quadratic regression includes an intercept and two terms: temperature (temp) and temperature squared (temp.sq).

Parameter	Estimate	SE	*P* value
(Intercept)	−2.671	0.461	<0.001
temp	0.399	0.058	<0.001
temp.sq	−0.011	0.002	<0.001

**Table 3:** Results of likelihood-ratio tests to evaluate improvements in model fit. Likelihood-ratio tests evaluated the evidence that potential variables improve model fit to simulate peak density of *C. shasta* compared to a published model containing only the prevalence of infection of hatchery-released Chinook Salmon from the previous spring (HPOI; Robinson et al., 2020). Abbreviations for variables are described in Table 1; *P* values ≤ 0.05 are indicated by an asterisk. The degrees of freedom for all tests is 1.

Model variables	*χ* ^2^	*P* value
HPOI + FF	5.380	0.020*
HPOI + precip	3.415	0.065
HPOI + Q.spsum	1.837	0.175
HPOI + T.spsum	1.672	0.196
HPOI + reserv	1.161	0.204
HPOI + T.sppeak	1.321	0.250
HPOI + Q.sppeak	0.287	0.592

The *χ*^2^ goodness-of-fit test for this model did not indicate any evidence of lack of fit (*P* = 0.120). For this model, there was strong evidence that HPOI (*P* < 0.001) and the occurrence of a flushing flow (*P* = 0.003) were associated with the spring peak of spore densities (Table 4). These results are also consistent with the ecological hypotheses suggesting consideration of these variables in this modeling exercise, as higher HPOI values are correlated with increases in annual peak spore densities, and years with flushing flows are simulated to result in lower annual peak spore densities (Table 4). After accounting for whether years had a flushing flow or not, every 10% increase in HPOI is estimated to increase peak spore density by 102% (95% CI [48–175]% increase). After accounting for HPOI, years with flushing flows are simulated to have peak spore densities that are 90% lower (95% CI [56–98]% lower) than years without flushing flows.

**Table 4:** Results of the negative binomial generalized linear model to predict the peak spore density. This model includes the prevalence of infection of hatchery-origin Chinook Salmon (HPOI) from the previous year (Robinson et al., 2020), and an indicator of flushing flow (FF; ≥171 m3/s).

Parameter	Estimate	SE	*P* value
HPOI	0.070	0.016	<0.001
FF indicator	−2.34	0.774	0.003
Dispersion	0.872	0.350	

### Spore density model

The model of *C. shasta* generally captured the onset of spore release, seasonal pattern of spore density, and inter-annual variation (Fig. 7), with sufficient contrast to inform management decisions. Most importantly, it closely matches observed values from the onset of spore release and initial increase in spore density for years in which density—and therefore disease risk for juvenile salmonids—was high. The model over- and under-predicted the lowest and highest (respectively) peak spore density values observed (Fig. 5D; *r* = 0.770), which is common for regression models (Faraway, 2006). For example, the model slightly overestimates spore density in 2011–2013 however both simulated and observed spore densities in these years were <10 spores/L (*r* = 0.390–0.437) indicating that both simulated and observed spore densities suggest lower disease risk for these years. For years in which spore densities were >10 spores/L, the goodness-of-fit for simulated values compared to observed densities was higher (*r* = 0.561–0.885).

## Discussion

Modeling the density of the waterborne stage of a parasite requires understanding the dominant drivers of the parasite’s complex lifecycle. Over a decade of monitoring data, sentinel fish studies, and laboratory experiments have identified how water temperature and discharge influence *C. shasta* densities in river water and how *C. shasta* densities impact juvenile Chinook and Coho salmon in the Klamath River (*e.g.*, Fujiwara et al., 2011; Hallett & Bartholomew, 2012; Ray & Bartholomew, 2013; Ray et al., 2015). The waterborne spore model presented here is informed by these empirically-derived relationships and includes the three components of *C. shasta* spore dynamics we aimed to simulate as important metrics in predicting disease risk (Fig. 4). The model combines both mechanistic (process-based) and statistical (correlative) relationships that are driven by variables that are sensitive to management decisions in this river system. Being responsive to management actions such as flushing flows makes the model useful for simulating disease risk for salmonid juveniles traveling through the infectious zone during their spring emigration.

Models using a combined approach (mechanistic and statistical) have been applied successfully in other parasite systems with aquatic transmission and both vertebrate and invertebrate hosts. Another myxozoan parasite, *T. bryosalmonae*, which causes Proliferative Kidney Disease (PKD) in salmonids, has been modeled successfully by incorporating the role of temperature and disease dynamics in hosts (Carraro et al., 2016). Schistosomes that cause disease in humans have been modeled using a thermal response and statistical relationships to estimate abundance of the parasite (Mangal, Paterson & Fenton, 2008; Stensgaard et al., 2016). Using a mechanistic approach to model parasites encapsulates complex transmission and lifecycle dynamics and can be useful to explore the impacts of disease under scenarios of climate change, drought (*e.g.*, Kearney et al., 2009; Kirk et al., 2018), or other changes in the physical environment. The modular approach and degree-day model used here can provide the framework for more complex disease models while we seek to understand the transmission mechanisms of *C. shasta*.

For some stages of the *C. shasta* lifecycle, we lack clear understanding of specific mechanisms driving their dynamics, and empirical studies have yet to yield definitive results. Adult salmon carcasses are a source of myxospores that infect annelid hosts in the infectious zone, but attempts to predict myxospore load have found no trend with fish age or sex (Foott et al., 2016) and no association has been detected between spawner abundance and spore densities the following spring (Robinson et al., 2020). The prevalence of infection of immunosuppressed adult fishes may not be as relevant to myxospore contribution as the severity of infection, where a small percentage of heavily infected carcasses contribute the highest myxospore loads (Foott et al., 2016). For the annelid host, the relationship between myxospore dose and actinospore production is highly variable (reviewed in Alexander et al., 2015). In *C. shasta*, actinospores released from individual infected annelids range from 350 (Meaders & Hendrickson, 2009) to 10,000 (J. Alexander, 2019, unpublished data) in 24 h. Studies of other myxosporeans reported myxospore to actinospore ratios ranging from 1:0.2 to 1:47 (*Thelohanellus hovorkai* and *M. cerebralis*, respectively; Liyanage, Yokoyama & Wakabayashi, 2003; Markiw, 1986). The spore model presented here incorporates annelid infection indirectly, using spawn timing as the start of infection and the initiation of actinospore development, and using the prevalence of infection of hatchery-origin juveniles from the previous spring to predict parasite loading of annelids (Robinson et al., 2020). Though gaps exist in our understanding of some lifecycle linkages in *C. shasta*, the model successfully simulates patterns of spring spore density using a combination of process-based and statistical relationships based on monitoring data. Future research that identifies specific mechanisms of spore transmission to hosts and dose ratios can strengthen the *C. shasta* spore model.

We built this *C. shasta* model to simulate spore densities for a site within the infectious zone during the period of outmigration for juvenile salmon in the lower Klamath River, however phases of the parasite’s lifecycle are distributed both up- and downriver. Outmigrating juvenile salmon exposed to *C. shasta* in the infectious zone swim downstream, whereupon infected fish that die before reaching the ocean can release myxospores that may be consumed by annelid populations in the lower river (Benson, 2014). Infected annelids release actinospores that may infect adult salmon returning to spawn upriver. The release of myxospores from post-spawn carcasses in turn drives infection in upriver annelid populations that provide the source of spring actinospores. Though the contributions of myxospores by adult salmon are likely greater than the contributions by juveniles (Benson, 2014), infection in outmigrating fish likely plays an important role in perpetuating the lifecycle of the parasite. Understanding the spatial and temporal span of *C. shasta* is necessary to inform any expansion of spore density simulations to other sites along the mainstem Klamath River.

Environmental changes in the Klamath River have led to an imbalance in host-pathogen equilibrium of *C. shasta*. The parasite *C. shasta* and invertebrate host *M. occidentalis* are assumed to have co-evolved with the salmon species they infect in the Klamath River (Breyta, Atkinson & Bartholomew, 2019). This apparent co-evolution of the parasite and its salmonid and annelid hosts should persist over time at a relatively low-level virulence equilibrium, given relative consistency in the environmental conditions in which this equilibrium evolved (Toft & Aeschlimann, 1991; Esch & Fernandez, 1993). However, significant alteration of environmental conditions, such as abrupt changes in hydrology, sediment balance, and water temperature regimes, among others, have occurred in this system from the development of agricultural water withdrawals in the Upper Klamath Basin and the construction of hydropower facilities on the Klamath River. These environmental changes provide conditions that benefit *C. shasta* and cause the parasite-host equilibrium to become out of balance. Shorter generation times of the parasite compared to that of the hosts may enable the parasite to adapt more quickly to major environmental shifts (Webster et al., 2007). These altered conditions also support the abundance of annelid hosts that indirectly benefits the parasite. This imbalance may be expressed as elevated infection levels in the host organisms over naturally-occurring background levels, consistent with the high infection levels observed in juvenile Chinook Salmon populations in the lower Klamath River downstream of Iron Gate Dam.

This spore model can be used in evaluating effects of management actions on disease-caused mortality of juvenile salmon by simulating spore density with or without flushing flows and with low or high HPOI. It also has applications as a submodel in other management tools that assess the impact of proposed flow regimes. In the S3 model, spore density along with inputs of water temperature and travel times through the infectious zone are used to predict how abundance and survival of juvenile salmon vary in relation to environmental conditions that can be impacted by flow regime characteristics. The spore model could also be used in a mixture cure model (Ray et al., 2014) to estimate mortality rates under difference management scenarios.

The modular approach we used to simulate spore density of *C. shasta* in the spring is advantageous because it can be easily adapted with new data or relationships as we learn more about specific mechanisms of the disease lifecycle. In particular, the inclusion of annelid monitoring data in the model may inform actinospore dose, however additional years of data are needed. Additionally, both laboratory studies and annual in-river monitoring are necessary to improve our understanding of dose and transmission. Differences between measurements of spore density collected using two different sampling techniques (manual and automatic sampler) are being evaluated in future studies. Exploring the mechanisms of transmission dynamics can be accomplished in laboratory studies where flow, temperature, and dose are controlled (*e.g.*, Bjork & Bartholomew, 2009), while population dynamics and infection rates are important to measure *in situ* in order to capture the inherent variability. Model inputs are based on the lifecycle of fall-run Chinook Salmon (juvenile POI, spawning estimates), however the total density of spores in the spring can also affect other salmonids (Atkinson & Bartholomew, 2010). Each species has different disease risk, spore thresholds, and mortality rates; the proportion of relevant *C. shasta* genotype would also need to be taken into account to adapt the model specifically for other salmonids.

## Conclusions

The spore model presented here can serve as an important decision support tool to assess the effects of potential management scenarios on disease risk to anadromous salmonids. For over a decade, the impacts of *C. shasta* on outmigrating salmon in the Klamath River have been a major focus of management actions that use flow modification to disrupt various aspects of the *C. shasta* lifecycle (NMFS & USFWS, 2013; NMFS, 2019). Flushing flows that can scour annelids from habitats and disturb the sediment and river bed are embedded into the spore model: the occurrence of a flushing flow delays the initial onset of spores, and strengthens the model fit to simulate peak spore density. Juvenile salmon mortality caused by *C. shasta* occurs in other highly regulated watersheds (Stinson & Bartholomew, 2012). Our work in the Klamath River provides a useful template for other watersheds, highlighting how mechanistic models of the pathogen lifecycle can be combined with analysis of long-term monitoring data to generate models relating key flow management variables to disease risk.

## Supplemental Information

10.7717/peerj.13183/supp-1 Supplemental Information 1 Raw data and code for analyses Measurements of Ceratonova shasta spore density collected at a monitoring site (Beaver Creek) along with daily river temperature (River Base Model 10 output at Beaver Creek) and daily river discharge (at Iron Gate Dam) in “spore_obs.csv”. Annual indices of biological and environmental conditions from publicly available reports are summarized in “modelvariables.csv” to test model fit.
The R code analyzing these data and producing figures is available in “robinson_sporemodel_inreview.R”. The R code demonstrates how the model was constructed, detailing each module A–C and how these three modules are combined in the final spore model simulating C. shasta spore density in the spring during outmigration of juvenile salmonids. Module A simulates the onset week when C. shasta is first detected above 1 spore/L in water samples using a bio-physical degree-day model. Module B simulates the seasonal pattern of spore density using daily water temperature and a temperature-dependent quadratic model. Module C simulates the magnitude of peak spore density in the spring using a generalized linear model.
The final model simulates spore density for weeks 1–30 in 2007–2018. For each year, the model simulates the week of spore release (A), normalized spore densities for days following the onset of spores (B), and an annual peak (C). Spore density is calculated by multiplying the simulated normalized spore density and peak spore density.

## Additional Information and Declarations

### Data Availability

The following information was supplied regarding data availability: The raw data and code are available in the Supplementary File and at ServCat: Robinson HE. 2021. Code and data for a model to predict density of *Ceratonova shasta* actinospores in the Klamath River. https://ecos.fws.gov/ServCat/Reference/Profile/132191.
